# Bacteriological and Physicochemical Quality of Drinking Water in Wegeda Town, Northwest Ethiopia

**DOI:** 10.1155/2021/6646269

**Published:** 2021-02-08

**Authors:** Baye Sitotaw, Eshetie Melkie, Denekew Temesgen

**Affiliations:** ^1^Department of Biology, Bahir Dar University, Bahir Dar, P.O. box 79, Ethiopia; ^2^Simada District Educational Office, Wegeda town, Simada, Ethiopia

## Abstract

Waterborne diseases continue to challenge communities in low-income countries like Ethiopia. Clinical information in Wegeda town showed that the prevalence of waterborne diseases was 58%. This study aimed to evaluate bacteriological and physicochemical drinking water quality in Wegeda town. This study will add valuable scientific data for future intervention. Water samples from protected and unprotected springs, hand-dug well, taps, and households' containers were collected from November 2018 to June 2019 for bacteriological and physicochemical analyses. Besides, information about the potential risk factors was collected using a structured questionnaire. A total of 120 water samples were collected and analyzed for total and fecal coliform counts using the multiple tube fermentation method (MPN). The presence of *Escherichia coli* was also checked from fecal coliform positive samples collected from households' containers. Selected physicochemical parameters were also determined using the standard methods. In all cases, the median values of total and fecal coliform counts ranged from 5 to 27 and 2 to 13 MPN/100 ml, respectively. Accordingly, all of the drinking water samples did not comply with the standards. Coliforms were significantly higher in the households' containers than in the sources (*p* < 0.05) and also significantly varied by water sources. The highest and lowest coliform counts were recorded in unprotected spring and taps, respectively. Besides, 18.33% of water samples collected from households' containers were tested positive for *E. coli*. Regarding physicochemical parameters, most values were within the acceptable limit values recommended by the WHO. However, water samples from unprotected spring and hand-dug well did not satisfy the turbidity limit value set by the WHO. Drinking water systems in Wegeda town were likely contaminated with pathogenic bacteria likely due to poor protection and sanitation practices. Providing the community with potable water, toilets, domestic and animal waste disposal systems, and intensive health education and sanitation practices for the community are highly recommended.

## 1. Introduction

Drinking water pollution has been a global challenge and poses a serious threat to human health. Drinking water can be polluted at the source, distribution line, and/or at the household level, and such polluted water can be vehicles for several pathogens [1, 2]. This problem is severe in poor societies due mainly to a lack of awareness about sanitary measures at the different levels.

Based on the WHO estimate, up to 80% of all illnesses and diseases worldwide are caused by waterborne and/or water-related diseases. Globally, an estimated 785 million people use unimproved water sources; some 144 million people rely on surface water for drinking, and more than 2 billion people use drinking water contaminated with feces [1]. Moreover, in low- and middle-income countries, a significant proportion of health care facilities lack improved water sources, improved sanitation, and lack water and soap for handwashing [3].

To achieve Goal 6 of SDG, providing adequate and quality water has been a priority area in Ethiopia. Accordingly, Ethiopia has met the target of 57% of the population using safe drinking water by 2015 [4]. Despite the considerable expansion of the water supply systems on account of reaching this goal, Ethiopia is still among the countries with the lowest basic water services and high prevalence of water-related diseases. Access to potable water and sanitation services in Ethiopia is among the lowest in sub-Saharan Africa. For example, safe drinking water coverage is about 66% [5], and only 6.3% of households have access to improved sanitation [6]. This problem is especially worst for rural people, who make up more than 80% of the Ethiopian population. More importantly, the majority of the rural households do not have sufficient understanding of hygienic practices regarding food, water, and personal hygiene. Moreover, only 17% of people practice improved hygiene behaviors and live in healthy environments [6].

In Ethiopia, limited access to safe water and inadequate sanitation and hygiene services accounted for 60 to 80% of communicable diseases and an estimated 50% of children's undernutrition. Poor sanitation, such as open defecation, can lead to stunting. Open defecation, for instance, can lead to fecal-oral diseases such as diarrhea, which in turn cause and worsen malnutrition. Diarrhea is the leading cause of under-five mortality in Ethiopia, accounting for 23% of all under-five deaths, that is, more than 70,000 children a year [6].

Several studies have been conducted regarding the bacteriological quality of drinking water in the different localities of Ethiopia. For instance, a study in Jimma zone (south west Ethiopia) by Yasin et al. [7] reported that all drinking water samples were tested positive for total and fecal coliforms; Tabor et al. [8] reported that 77% of the drinking water samples in Bahir Dar city (Northwest Ethiopia) were tested positive for total coliform counts, and they had a high-risk score; a study by Berhanu and Hailu [9] in Bona District (southern Ethiopia) showed that 86% of the drinking water did not meet the WHO guidelines for drinking water qaulity; a study in Adama town (Eastern Ethiopia) by Temesgen and Hameed [10] showed that 44.2% and 28.9% of the water samples were tested positive for fecal coliforms and fecal streptococcus, respectively; Feleke et al. [11] reported fecal coliform count of 82.1 to 86.8% in drinking water in Wogera town (Northern Ethiopia); Duressa et al. [12] reported total coliform count of 100% and fecal coliform count of 37% in drinking water sample in Nekemte town (Western Ethiopia); a study by Alemayehu et al. [13] in the southern region showed that 44.7% and 50.9% of the drinking water samples were contaminated with *E. coli* and *Enterococcus*, respectively, and had a high-risk score; and a study by Gizachew et al. [14] in Boloso Sore district (southern Ethiopia) reported that 91% and 44% of drinking water samples collected from households and the sources were tested positive for fecal coliforms.

The populations of Wegeda town obtain drinking water from one borehole through the pipe, five hand-dug wells, one protected spring (direct or through the pipe), and several unprotected springs (direct). The source of tap water at an individual house was thus both a protected spring and a borehole. According to official reports from the health sector of the town, waterborne diseases were among the top ten causes of illness in the community. For instance, in 2018 alone, 58% (11,076/19,096) of the patients visiting the health centers were sick due to typhoid fever, dysentery, or diarrhea. However, no scientific study was conducted in the current study area, and this study was thus aimed to evaluate the bacteriological and physicochemical quality of drinking water in Wegeda town (South Gondar, Amhara region, Ethiopia) using standard procedures. The result of this study will reveal the extent of problems related to drinking water pollution and hence influence concerned bodies for appropriate intervention and trigger scientific communities for further studies on the problem.

## 2. Materials and Methods

### 2.1. Study Area Description

This study was conducted in Wegeda town, Simada district, South Gondar Zone, Amhara region, Ethiopia. This town is situated some 774 km north of Addis Ababa, the capital city of Ethiopia. The town is located at the latitude of 11° 23′ 52.69″ N, longitude of 38° 14′ 16.11″ E, and an elevation of 2555 meters above sea level. The town has a climatic zone of Woynadega (midland), where 5% of the population in the Simada district lives in this town. The primary wet season extends from April to October, where July and August are the wettest months. The mean annual rainfall ranges from 900 to 1100 mm and the mean annual temperature is about 23°C.

Based on the official report of Wegeda town administration offices, the town has an estimated total population of 16, 282 of which 7978 were male and 8304 female. There were a total of 4224 households of which 3,787 were male-headed and 137 female-headed. According to the information from the Wegeda town water office, there were improved drinking water sources (one borehole, one protected spring, and five hand-dug wells) and four unprotected springs. Although 68% of the Wegeda town population receives water from improved sources, water coverage (quantity per person) remains very low (43% in the year 2018).

### 2.2. Study Design and Parameters

A cross-sectional study was conducted in Wegeda town from November 2018 to June 2019 to assess the microbiological and physicochemical quality of drinking water. Sampling sites were selected based on the availability (purposively) of the different types of water sources for drinking purposes. Water samples were thus collected from three sampling points at the source hand-dug well (HDW), protected spring (PS), and unprotected spring (UPS). In addition, water samples were collected from piped water (TW) at selected individual taps as well as from households' containers (H) using clean and/or sterilized containers for both bacteriological and physicochemical analyses (Table 1).

Bacteriological water quality indicator organisms, namely, counts of total coliforms (TCC) and fecal coliforms (FCC) using multiple fermentation tubes method, and detection of *Escherichia coli* using selective media were conducted. Selected physiochemical parameters, namely, turbidity, pH, temperature, electrical conductivity, NO_3_^−^, SO_4_^2−^, and NH_3_, were determined following standard methods [15]. In addition, visual assessment of the water sources (using observational checklist) and structured questionnaire were conducted to assess the sanitary status and risk factors for water quality deterioration.

### 2.3. Sample Size, Sampling, and Sample Handling

A total of 120 water samples (from 15 taps, 15 hand-dug wells, 15 protected springs, 15 unprotected springs, and 60 households' containers) were collected from urban sites of Wegeda town for bacteriological examination and physicochemical analyses. Two hundred and fifty milliliters of water sample was collected aseptically with sterilized bottles both from the sources and households' containers for bacteriological. Samples for chemical analyses were collected with acid-washed one-liter polyethylene bottles. The samples were taken in the morning between 7.00 and 8.00 a.m. Water samples were then kept in an icebox and transported to the microbiology laboratory within 4 hours. The analyses were begun immediately after the sample had arrived at the microbiology laboratory, Bahir Dar University, Ethiopia.

### 2.4. Bacteriological Quality Analysis of Water Samples

#### 2.4.1. Enumeration of Coliforms and *E. coli*

Coliform enumeration was done using the Most Probable Number technique (MPN). The method, in brief, was as follows: fifteen culture tubes were used per sample where five tubes contain sterile 10 ml double strength and ten tubes contain 10 ml single strength Lauryl tryptose broth (Blulux Laboratories Ltd., India), all tubes with inverted Durham tubes. With a sterile pipette, 10 ml of the water sample was aseptically dispensed into each of the first five culture tubes containing the double strength Lauryl tryptose broth. Into the rest of the ten tubes containing sterile single strength Lauryl tryptose, 1 ml of the sample was inoculated into each of the five culture tubes, while 0.1 ml sample was inoculated into the remaining five tubes all with inverted Durham tubes. The tubes were gently shaken to distribute the sample uniformly throughout the medium and incubated at 37°C for 24 hours. After 24 hours of incubation, the cultures were observed for color change (acid production) or gas formation. For the confirmatory test, a loop full of culture from test tubes that showed gas production was transferred to the brilliant-green lactose bile (BGLB) broth tube and incubated for 24 to 48 hours at 35°C. Tubes showing gas and growth were considered as positive for total coliforms (TC). Finally, results were reported as the Most Probable Number per 100 milliliters of water sample (MPN/100 ml). The same procedure was followed for fecal coliforms (FC) except that the tubes were incubated at 44.5°C for both presumptive and confirmatory tests. The presence of *E. coli* was cheeked from FC positive tubes streaked on Eosin–Methylene Blue plate and incubated for 24 hours at 37°C. The presence of golden greenish shiny color was taken as evidence for the presence of *E. coli*.

### 2.5. Determination of Physicochemical Parameters

Temperature (using Bio Abron Student Mercury Thermometer, pH (using Wagtech pH meter, model CP 1000 Singapore), turbidity (using Wagtech turbidity meter (model Wag–WT 302, Singapore), and TDS and conductivity (using TDS/Conductivity meter, Wagtech 534000, Singapore) were measured *in situ.* The concentrations of the following major ions were determined within 4 hours after collection and immediately after arrival to the laboratory following standard methods of water and waste examination [16]. Nitrate (NO_3_^−^), sulphate (SO_4_^2−^), and ammonia (NH_3_^−^) were determined by photometric methods using Palintest Photometer 7100 (Wagtech, Thatcham. Berkshire, UK).

### 2.6. Sanitary Assessment

The assessment of the sanitary status of the drinking water sources was carried out through visual inspection following the checklist listed in the WHO guidelines for drinking water quality [17]. Structured questionnaires were also used to obtain information on sanitary integrity and the potential hazards in the environment that may affect drinking water quality.

### 2.7. Data Analysis

Data were analyzed using SPSS statistical software (version 20). The Shapiro–Wilk test was used to check the normality of the data. Significant differences in the water quality parameters (bacteriological and physicochemical) among the different drinking water sources and households' containers were tested using Mann–Whitney *U* test. Moreover, significant differences in the values of each parameter across the samples were checked using the Kruskal–Wallis test.

Values of bacterial counts and physicochemical parameters of the investigated water samples were compared with the standards (WHO guidelines or Ethiopian standards for drinking water quality) and interpreted as acceptable or unacceptable. In addition, water samples were categorized into the different risk levels according to risk classification for thermotolerant coliforms or *E. coli* of rural water supplies [16]. The association between independent variables (sociodemographic and other risk factors) and the dependent variables (values of bacteriological and physicochemical water quality parameters) were computed using Chi-square test. In all cases, statistical significance was considered at a 95% confidence interval and *p* values of ≤0.05.

### 2.8. Limitations of the Study

Due to various reasons, the following limitations were compromised. Drinking water quality analyses usually cover the sources, the reservoir (disinfection point), tap (point of use), and the storage container. In this study, however, samples were not taken from the reservoir which would have been better to evaluate the appropriate chlorination. In addition, all samples should have been tested for the presence of *E. coli* to approximately find out the possible source of contamination; residual chlorine should have been determined to check the proper treatment of the system; more numbers of hand-dug wells and unprotected springs should have been sampled to get a more conclusive result. Moreover, self-administered questionnaires usually bias the results.

## 3. Results

### 3.1. Bacteriological Quality of Drinking Water in Wegeda Town

In this study, 94.16% (113/120) of the drinking water samples were tested positive for total coliforms (TC), which means that they did not comply with the WHO guidelines [17]. Variation in the median counts of TC among the different water samples was significant (*p* < 0.05). The highest (27 MPN/100 ml) and the lowest (5 MPN/ml) median TC counts were recorded in the water samples taken from households' containers that used unprotected spring and water samples taken from the tap, respectively. Comparing the sources and the household containers, 85% (53/60) of the water samples from sources and 100% (all the 60 samples) from households' container, respectively, were positive for TC. There were statistical differences in the counts of TC at a water source and household's container (*p* < 0.05) irrespective of the type of water sources (Table 2). Regarding fecal coliforms (FC), 82.5% of the water samples were tested positive for FC and did not satisfy the WHO guidelines. Of these, 46.66% (7/15), 73.33% (33/45), and 98.33% (59/60) of the water samples from sources, taps, and households' container, respectively, were tested positive for FC. It should also be noted that there were statistical differences in the median counts of FC at a water source and household's container (*p* < 0.05) irrespective of the type of water sources except in the tap water samples (Table 2). Moreover, *Escherichia coli* was detected in 18.33% (11/60) of drinking water samples taken from households' containers.

According to the WHO [16] risk classification for thermotolerant coliforms, all water samples from households' containers and unprotected spring, 80% of water samples from protected spring, and 66.66% of water samples from tap and hand-dug well fell into the intermediate risk category. The rest of the samples fell into the low risk category.

### 3.2. Physicochemical Drinking Water Quality

Measured values for most of the physicochemical quality of drinking water in Wegeda town were found to be within the permissible limits set by the WHO, except for median temperature of all samples, turbidity of water samples from unprotected spring (both from the source and households' containers), and households' containers fetched from hand-dug well (Table 3). Median temperature value ranged from 16.1 (TW-H sample) to 24°C (UPS sample), while median turbidity value ranged from 1.5 (TW sample) to 8.9 NTU (UPS-H sample).

Maximum median pH and temperature, respectively, were recorded in HDW (7.27) and UPS (24), while the minimum median pH and temperature were recorded in water samples from unprotected spring (6.51) and households' containers fetched from tap water (16. 1°C), respectively. Most of the water samples from TW and HDW, water samples from PS-H, and water samples from HDW-H (13/15) satisfied the WHO guidelines. Similarly, far more than half (10/15) of water samples from PS and water samples from UPS-H and about half (8/15) of water samples from UPS had pH values within the limits set in national and WHO guidelines.

In the present study, the minimum and maximum median conductivity values (*μ*s/cm) were recorded in UPS (441) and TW (762 *μ*s/cm) (Table 3). Generally, conductivity values were higher in TW (both directly from the tap and tap water from households' container) compared to water samples from springs and hand-dug wells (still both directly from the sources as well as from households' container).

From a total of 15 water samples from each of HDW, PS and PS-H, UPS and HDW-H, and UPS-H 4 (26.6%), 7 (46.6%), 10 (66.6%), and 13 (86.6%) were above the recommended WHO limit for turbidity. However, all water samples from TW and TW-H were found to satisfy the guideline value (Table 3). Regarding the ions, the values of nitrate, ammonia, and sulphate from all types of water sources and households' containers were found to be within the standard limit set by the WHO.

### 3.3. The Risk Factors at the Household Level and Association with Fecal Coliform Counts and *E. coli* Contamination

A total of 60 households (HHs) living in Wegeda town were included in this study. All respondents from these HHs were females owing to their high responsibilities related to drinking water. As indicated in Table 4, most of the respondents (households) were between 31 and 50 years old, completed primary school, were government employees, earned Ethiopian Birr 3001–4000 monthly, and had a family size of above 5.

The risk factors included in this study and their association with drinking water contamination are indicated in Table 4. Among the 60 households interviewed and or inspected, 23 (38.3%) of them had no toilet, and even most of the available toilets were almost nonfunctional. Regarding the behavior of the participants, 21 (35%) of them did not wash their hands after using the toilet. In addition, 34 (56.7%) of the participants draw water by dipping cups into the storage containers. Concerning the knowledge of the participants about drinking water handling, half of the respondents did not have information about water handling practice. Moreover, 33 (55%), 14 (23.3%), and 41 (68.3%) of them did not know that unclean containers, uncovered containers, and unsanitary hands, respectively, can contaminate drinking water.

Among the thirteen factors, educational status and occupation of the family head, family monthly income, source of drinking water, presence of toilet, handwashing habit after using toilet, and knowledge about the fact that uncovered container can contaminate drinking water had a strong association with FCC and *E. coli* contamination (*p* < 0.05).

## 4. Discussion

Public health burden due to low drinking water quality has been common in low-income countries like Ethiopia. The population in Wegeda town is not an exception to this problem. This study aimed to assess the microbiological and physicochemical quality of drinking water and some associated risk factors in Wegeda town. Water samples from purposely selected sources (a hand-dug well and two springs) and sixty randomly chosen households (from both taps and storage containers) were tested for the aforementioned water quality parameters.

### 4.1. Bacteriological Quality of the Water Samples

The World Health Organization (WHO) as well as Ethiopian guidelines for drinking water quality does not allow any detection of coliforms or *E. coli* in 100 ml of drinking water. In the present study, however, most water samples were likely contaminated with bacteria pathogens as coliforms were detected in most of the samples (total coliform counts, 94.16%; fecal coliforms, 82.5%) and found to exceed the recommended values in the WHO and national standards [17, 18].

In all cases, water samples collected from households' containers had a higher level of contamination compared to the sources and the taps, and the differences were statistically significant (*p* < 0.05) (Table 2). Almost all of the households were at risk of waterborne diseases likely due to improper handling practices of drinking water at home. This strongly suggests a lack of knowledge and handling practices of drinking water at the household level.

Regarding the water sources, the highest median value of total coliform counts and fecal coliforms counts were recorded in the water sample collected directly from an unprotected spring. The higher level of contamination in the unprotected springs is likely due to poor protection and exposure to contamination by wastes from humans, animals, and the surrounding environment.

A high count of fecal coliforms in drinking water is direct evidence for fecal contamination and the occurrence of waterborne diseases in the community. Even though a high count of total coliforms may not directly show pathogenic bacteria contamination, this at least indicates chlorination has not been done properly, which in turn implies pathogenic bacteria contamination of the drinking water.

In this study, *Escherichia coli* was detected at a relatively lower rate compared to fecal coliforms. However, as far as public health is concerned, *E. coli* contamination never is compromised as this test provides strong evidence of recent fecal contamination [19]. It is always found in feces and is, therefore, a more direct indicator of fecal contamination and the possible presence of enteric pathogens. More importantly, *E. coli* serves as a member of fecal coliforms and as an index for *Salmonella* contamination and is more stable when compared with TC.

Similarly, studies conducted in the different localities of Ethiopia such as in Bahir Dar town [8], Dire Dawa [20], north Gonder [21], Jimma town [7], Sidama Zone [9], Shambu town [22], and Nekemte town [12] have shown a high level of coliform counts that did not meet the WHO permissible level. This implies that bacterial contamination of drinking water continues to be a public health concern in Ethiopia.

### 4.2. Sanitary Conditions of the Water Sources and the Households

Water supply alone, in the absence of proper sanitation and hygienic condition, can be meaningless. The sanitation coverage in Wegeda town during 2012 was reported to be 49.5% [23], which is far lower than the national plan set to reach 66% by 2015.

The sanitary condition around the water sources and drinking water handling practices in Wegeda town were generally poor. This is supported in that a significant proportion of the community lack toilet had poor knowledge and drinking water handling practices. Education and poverty-related factors such as types of occupation, income, source of drinking water, and access to the toilets were found to be aggravating factors for the observed low drinking water quality in Wegeda town.

### 4.3. Physicochemical Drinking Water Quality

Generally, the physicochemical drinking water quality in Wegeda town was acceptable. Relatively, conductivity was higher in tap water than spring and HDDs. This might be due to the chlorination of tap water or problems related to water containers. However, the temperature of drinking water samples was around the optimum growth condition for the proliferation of aerobic mesophilic bacteria and, thus, could contribute to the high bacterial counts observed. The mean turbidity of water samples from UPS was the highest of all records suggesting that this spring was exposed to polluting activities in the vicinity, and, thus, it is unsafe to drink from it. Similarly, water samples from the hand-dug well (at both the source and households' container) had a higher mean value of turbidity suggesting the presence of some factors around the water point, However, all water samples from taps (directly from tap and households' container) had an acceptable value of turbidity. Also, the mean values of nitrate and ammonia were lower in TW compared to values in other samples, which could indicate at least the physical water purification system was properly working.

## 5. Conclusions

The result of this study showed that all water samples fail to comply with the WHO or national standards for drinking water quality in terms of coliforms. A significant proportion of water samples were also contaminated with *E. coli*. However, the physicochemical parameters in all drinking water samples were within the permissible limit of international guidelines, except for turbidity and temperature. The risk factors for water quality deterioration including educational status, occupation, family income, source of drinking water, access to quality toilets, and drinking water handling practices were found to associate with bacterial contamination. As a long-term solution, education and poverty alleviation strategies would be crucial to the Wegeda town community in order to reduce the prevailing water-related health burden. We recommended that proper chlorination of the drinking water system, regular monitoring of the water quality, provision of toilets and waste disposal systems, and intensive health education and sanitation practices for the community should be urgent tasks for concerned bodies.

## Figures and Tables

**Table 1:** Drinking water sampling scheme for bacteriological and physicochemical quality assessment in Wegeda town, 2018/19.

Water source	Code	Number of samples	Descriptions
Protected spring	PS	15	Directly from the spring (source)
	PS-H	15	From households' containers using this protected spring directly
	TW	15	From tap piped from this spring
	TW-H	15	From households' containers using the tap piped from this protected spring

Unprotected spring	UPS	15	Directly from this unprotected spring (source)
	UPS-H	15	From households' containers using this unprotected spring directly

Hand-dug well	HDW	15	Directly from the well (source)
	HDW-H	15	From households' containers using this well directly

**Table 2:** Comparison of median count (*n* = 15), ranges, and mean rank of total and fecal coliform bacteria (MPN/ml) in the different water sources and households' container in Wegeda town, 2018/19.

Bacteria	Samples	Median (SE^*∗*^)	Range	Mean rank	Mann–Whitney *U* test	*p* value (asymp. sign)
TC	TW	5.00 (1.25)	2–13	9.97	29.5	0.001
	TW-H	13.00 (1.82)	6–26	21.03		
	HDW	5.00 (1.77)	2–21	10.70	40.5	0.003
	HDW-H	13.00 (3.62)	6–46	20.30		
	PS	11.00 (2.26)	2–33	12.17	62.50	0.038
	PS-H	17.00 (4.22)	4–52	18.83		
	UPS	11.00 (3.48)	4–39	10.77	41.5	0.003
	UPS_H	27.00 (8.61)	11–110	20.23		

FC	TW	2.00 (0.74)	2–7	10.5	37.5	0.002
	TW-H	7.00 (1.34)	2–17	20.5		
	HDW	5.00 (0.90)	2–11	10.77	41.5	0.002
	HDW-H	9.00 (1.63)	5–21	20.33		
	PS	4.00 (0.84)	2–11	10.90	43.5	0.003
	PS-H	7.00 (1.56)	4–21	20.10		
	UPS	6.00 (1.44)	2–15	11.13	47	0.006
	UPS_H	13.00 (3.20)	4–39	19.87		

Note: ^*∗*^standard error of the median. TC = total coliform count; FC = fecal coliform count; sample codes are as indicated in Table 1.

**Table 3:** Median (range) (*n* = 15) values of the physicochemical parameter in the drinking water samples from the different sources and households' containers in Wegeda town, 2018/19.

Samples	pH	Temperature (°C)	Turbidity (NTU)	Conductivity (*μ*s/cm)	NO_3_ (mg/l)	NH_3_ (mg/l)	SO_4_ (mg/l)
TW-H	7.11 (1.32)	16.1 (5.81)	2 (2.2)	749 (68)	5 (7.71)	0.02 (0.11)	3.4 (3.7)
PS-H	6.8 (2.04)	20.4 (1.1)	5 (2.8)	513 (160)	6.02 (16)	0.3 (0.31)	4 (3.09)
HDW-H	6.6 (2)	20.8 (3)	7 (14)	616 (45)	6.6 (15.15)	0.18 (0.57)	3.55 (4.1)
UPS-H	6.6 (2.17)	21 (4.37)	8.9 (22.1)	493 (61.8)	13.22 (14.17)	0.12 (0.36)	3.55 (4)
PS	6.56 (2.09)	22 (7.5)	5 (3)	512 (114)	6 (22.01)	0.2 (0.7)	3.65 (3.1)
UPS	6.51 (2.08)	24 (6)	7 (20.5)	441 (96.5)	12 (8.1)	0.32 (0.37)	5.3 (5.78)
TW	7.12 (1.93)	17 (4)	1.5 (1.4)	762 (40)	3.9 (9.77)	0 (0.31)	3.01 (2.5)
HDW	7.27 (0.84)	23 (5.8)	4 (26)	632 (30)	7.1 (16.5)	0.11 (0.35)	3.3 (3)
WHO	6.5–8.5	<15	<5	1000	50	1.5	250

Note: sample codes are as indicated in Table 1.

**Table 4:** Association between the level of bacterial contamination (fecal coliform counts and presence of *E. coli)* of drinking water with sociodemographic characteristics of the participant and some other risk factors in Wegeda town, 2018/19.

Risk factors	Categories	Intermediate risk (%)	Low risk number (%)	Total number (%)	*χ*2 (*p* value)
*Sociodemographic characteristics of the households*					
Age of the respondents	18–30	2 (18.18)	9 (81.80)	11 (18.3)	3.73 (0.155)
	31–50	17 (51.51)	16 (48.48)	33 (55)	
	>50	7 (43.75)	9 (56.25)	16 (26.7)	
Educational status of the family head	No schooling	11 (78.57)	3 (21.42)	14 (23.33)	9.96 (0.019)
	Primary school	10 (34.48)	19 (65.51)	29 (48.33)	
	Secondary school	4 (36.36)	7 (63.63)	11 (18.33)	
	College and above	1 (16.66)	5 (83.33)	6 (10)	
Occupation of the family head	Farming	10 (90.90)	1 (9.10)	11 (18.33)	13.04 (0.005)
	Daily Laborer	4 (44.44)	5 (55.55)	9 (15)	
	Gov. employee	7 (30.43)	16 (69.56)	23 (38.33)	
	Merchant	5 (29.41)	12 (70.58)	17 (28.33)	
Family income in ETB Birr	<2000	6 (100)	0	6 (10)	13.79 (0.008)
	2001–3000	6 (60)	4 (40)	10 (16.7)	
	3001–4000	9 (42.85)	12 (57.14)	21 (35)	
	4001–5000	3 (17.64)	14 (82.35)	17 (28.33)	
	>5000	2 (33.33)	4 (66.66)	6 (10)	
Family size	2–4	11 (52.38)	10 (47.61)	21 (35)	4.46 (0.108)
	5	3 (20)	12 (80)	15 (25)	
	>5	12 (50)	12 (50)	24 (40)	

*Socioeconomic and behavioral factors*					
Source of drinking water	HDW	6 (40)	9 (60)	15 (25)	21.99 (<0.001)
	PS	3 (20)	12 (80)	15 (25)	
	UPS	14 (93.33)	1 (6.66)	15 (25)	
	TW	3 (20)	12 (80)	15 (25)	
Presence of toilet at home	Present	9 (24.32)	28 (75.67)	37 (61.7)	14.20 (<0.001)
	Absent	17 (73.91)	6 (26.08)	23 (38.3)	
Do you wash your hands after toilet with soap and water?	Yes	9 (23.07)	30 (76.92)	39 (65)	18.62 (<0.001)
	No	17 (80.95)	4 (19.04)	21 (35)	

*Knowledge on drinking water handling*					
Do you take water by dipping the cup into the storage container?	Yes	18 (52.94)	16 (47.05)	34 (56.7)	2.95 (0.086)
	No	8 (30.76)	18 (69.23)	26 (46.3)	
Have you ever had information about water handling practice?	Yes	12 (40)	18 (60)	30 (50)	0 .27 (0.60)
	No	14 (46.66)	16 (53.33)	30 (50)	
Do you know that unclean container can contaminate drinking water	Yes	18 (29.62)	19 (70.37)	27 (45)	3.75 (0.053)
	No	18 (54.54)	15 (45.45)	33 (55)	
Do you know that uncovered container can contaminate drinking water?	Yes	15 (32.60)	31 (67.39)	46 (76.7)	9.23 (0.002)
	No	11 (78.57)	3 (21.42)	14 (23.3)	
Knowledge that unsanitary hand can contaminate drinking water	Yes	5 (26.31)	14 (73.68)	19 (31.7)	3.27 (0.07)
	No	1 (51.21)	20 (48.78)	41 (68.3)	

## Data Availability

All data supporting the findings of this study are included in the paper; however, details of the full data may be obtained from the corresponding author on request.
